# Convex-Envelope Based Automated Quantitative Approach to Multi-Voxel ^1^H-MRS Applied to Brain Tumor Analysis

**DOI:** 10.1371/journal.pone.0137850

**Published:** 2015-09-14

**Authors:** Weibei Dou, Mingyu Zhang, Xiaojie Zhang, Yuan Li, Hongyan Chen, Shaowu Li, Min Lu, Jianping Dai, Jean-Marc Constans

**Affiliations:** 1 Department of Electronic Engineering, Tsinghua University, Beijing, China; 2 Radiology Department of Beijing Tian Tan Hospital, Capital Medical University, Beijing, China; 3 Beijing Neurosurgical Institute, Beijing, China; 4 Neuroradiology Service, University Hospital, Amiens, France; University of Pécs Medical School, HUNGARY

## Abstract

**Background:**

Magnetic Resonance Spectroscopy (MRS) can measure *in vivo* brain tissue metabolism that exhibits unique biochemical characteristics in brain tumors. For clinical application, an efficient and versatile quantification method of MRS would be an important tool for medical research, particularly for exploring the scientific problem of tumor monitoring. The objective of our study is to propose an automated MRS quantitative approach and assess the feasibility of this approach for glioma grading, prognosis and boundary detection.

**Methods:**

An automated quantitative approach based on a convex envelope (AQoCE) is proposed in this paper, including preprocessing, convex-envelope based baseline fitting, bias correction, sectional baseline removal, and peak detection, in a total of 5 steps. Some metabolic ratios acquired by this quantification are selected for statistical analysis. An independent sample t-test and the Kruskal-Wallis test are used for distinguishing low-grade gliomas (LGG) and high-grade gliomas (HGG) and for detecting the tumor, peritumoral and contralateral areas, respectively. Seventy-eight cases of pre-operative brain gliomas with pathological reports are included in this study.

**Results:**

Cho/NAA, Cho/Cr and Lip-Lac/Cr (LL/Cr) calculated by AQoCE in the tumor area differ significantly between LGG and HGG, with *p*≤0.005. Using logistic regression combining Cho/NAA, Cho/Cr and LL/Cr to generate a ROC curve, AQoCE achieves a sensitivity of 92.9%, a specificity of 72.2%, and an area under ROC curve (AUC) of 0.860. Moreover, both Cho/NAA and Cho/Cr in the AQoCE approach show a significant difference (*p*≤0.019) between tumoral, peritumoral, and contralateral areas. The comparison between the results of AQoCE and Siemens MRS processing software are also discussed in this paper.

**Conclusions:**

The AQoCE approach is an automated method of residual water removal and metabolite quantification. It can be applied to multi-voxel ^1^H-MRS for evaluating brain glioma grading and demonstrating characteristics of brain glioma metabolism. It can also detect infiltration in the peritumoral area. Under the limited clinical data used, AQoCE is significantly more versatile and efficient compared to the reference approach of Siemens.

## Introduction

### Background

Gliomas are a type of tumor arising from glial cells. Gliomas constitute about 80% of all malignant primary brain tumors [1–2]. The main biochemical characteristics can provide useful information on brain tumor type and grade noninvasively [3]. In many studies, *in vivo* Proton Magnetic Resonance Spectroscopy, abbreviated as ^1^H-MRS, has been shown to determine tumors type and grade [4–6]. According to the World Health Organization (WHO) grading system, low-grade gliomas (LGG) correspond to WHO grade I-II, and high-grade gliomas (HGG) correspond to WHO grade III-IV [7]. Low- and high-grade gliomas differ significantly with respect to biological behavior, growth pattern, growth rate, prognosis and other attributes. Furthermore, distinct strategies are adopted in clinical treatment. Therefore, differentiating between low- and high-grade gliomas accurately is extremely important, particularly when judging the grade of pre-operative brain tumors [8].

At present, the main noninvasive inspection method before surgical treatment of a brain tumor is neuroimaging, of which Magnetic Resonance Imaging (MRI) is the optimal choice. A conventional MRI exam depicts a structural image of the brain, with the advantage of providing precise anatomical information about the brain tumor. Interpreting the degree of malignancy exclusively from a morphologic point of view, MRI provides little helpful biological information on other aspects of gliomas. These aspects include grade, type, boundaries with normal brain tissue, metabolic characteristics of tumoral substances, and biological behavior. A large amount of information in addition to the anatomical structure is beneficial for a more comprehensive understanding brain tumors. As a result, this type of information has gained more clinical attention and has gradually become the key to differential diagnosis and prognosis of brain tumors. As the MRI technology advances, new magnetic resonance imaging technologies such as Magnetic Resonance Spectroscopy (MRS), Diffusion-Weighted Imaging (DWI), Diffusion Tensor Imaging (DTI) and Perfusion Weighted Imaging (PWI) have emerged, providing us with new approaches for recognizing, diagnosing and classifying brain tumors. Therefore, MRS, which can measure *in vivo* brain tissue metabolism and show its biochemical information, has been widely used [9–10]. Thus, these techniques, particularly ^1^H-MRS, can aid in brain glioma diagnosis, differential diagnosis, grading, and classification.

### Quantitative approach to multi-voxel ^1^H-MRS

Many studies have shown that the type and grade of brain tumors is related to the concentration of certain metabolite components [11–13]. The typical quantitative indicators of magnetic resonance spectroscopy observation are Choline (Cho), N-Acetyl Aspartate (NAA), Creatine (Cr), Lactate (Lac), Lipid (Lip) and other observational data which are all biochemical metabolites from brain tissue. When a brain tumor appears, the contents of these biochemical metabolites vary in a regular manner. By determining the regular patterns, then discovering metabolic properties of different types of gliomas, we can obtain some useful information for clinical prediction of gliomas. Since biomedical metabolite information is acquired through quantification of MRS data, proper and optimized quantification techniques should influence the objectivity and robustness of its result.

MRS quantification methods can be classified into time-domain and frequency-domain according to the data feature analysis method. Time-domain quantification is based on mathematical models. Since the expression of models, such as Lorentzian lineshape and Gaussian lineshape, is concise, computation can be efficient. Time-domain quantification methods are usually divided into two main classes: interactive methods and black-box methods. The former, making good use of prior knowledge, are flexible but difficult to quantify automatically. Black-box methods limit imposing prior knowledge for simplicity, thus improving the accuracy, and are also difficult. VARPRO [14] is the first widely used interactive quantification method. AMARES [15] later performed better in terms of robustness and flexibility. HLSVD [16] is a well-known black-box method with many variants. QUEST [17–18] is implemented in the software package jMRUI-3.0 (http://www.mrui.uab.es/mrui/), using linear fitting to quantify on the basis of a set of metabolite signals. Because of the poor signal to noise ratio of clinical ^1^H-MRS data, the advantage of some complex methods such as QUEST on accuracy is not clear.

The frequency-domain quantification is more intuitive and straightforward. Integrating the spectrum directly is convenient, but the overlap of some metabolite component peaks *in vivo* and unsatisfactory baseline correction may lead to large error. Therefore, quantification based on model fitting [19] is used more widely *in vivo*. Generally these methods assume that the real part of the spectrum fits Lorentzian, Gaussian or Voigt lineshape and that the baseline can be approximated as polynomials or splines. TLS [20] is just one representative of these methods, as is the LCModel [21]. The LCModel is similar to QUEST, fitting the spectrum to a linear combination of the metabolite basis set [22]. After considering the set as prior knowledge, results become more precise and need more time resources.

The Chemical Shift Imaging (CSI) technique can provide multi-voxel MRS in one examination, which results in much larger amounts of data to process. Some interactive quantification methods for Single Voxel Spectroscopy (SVS) such as AMARES, burdens the user with a large quantity of repetitive and unpleasant work. Furthermore, the quantitative result is difficult to reproduce because of differences in users’ prior knowledge and their state. Therefore, automated quantification is required to replace the interactive quantification with a more efficient and robust method.

In this paper, we present a new automated quantitative approach based on a convex envelope (AQoCE). This approach can be considered as a simulation of manual fitting, which can ensure efficiency and robustness, as well as give reasonably accurate results. AQoCE is a black-box method, so lacking physical meaning is still inevitable. To evaluate the performance of this new quantitative technique in grading the diagnosis of brain gliomas, AQoCE was applied to analyze the MRS data from 78 glioma cases acquired before brain tumor surgery, then the results were compared with their pathology reports. When compared to the MRS data processing software from Siemens (Trio Tim 3.0 T MRI’s workstation), our AQoCE approach provides results with higher specificity and sensitivity on these samples.

## Materials and Methods

### Materials

This research was approved by the medical ethics committee of Beijing Tian Tan Hospital, Capital Medical University. Written informed consent of each adult patient (participants) was obtained, and the written informed consents of the three minor patients’ guardian completed on behalf of the minor participants were also obtained. Data were obtained from 78 cases, including 48 males (10–73 years: average age, 44.71 years); 30 females (22–68 years: average age, 43.37 years), male/female, 1.6:1; all of the cases have been confirmed by pathology. All cases (n = 78) were classified into two groups: 1) 36 cases of low-grade gliomas (LGG, WHO I-II), including 18 astrocytoma, 5 oligodendroglioma, 12 oligdendro-astrocytoma, and 1 mixed neuro-glioma; and 2) 42 cases of high-grade gliomas (HGG, WHO III-IV), including 10 anaplastic oligdendro-astrocytoma, 7 astrocytoma, 24 glioblastoma, and 1 gliomatosis cerebri. All participants underwent non-contrast and contrast MRI and non-contrast MRS before surgery. See details in Table 1.

**Table 1:** Pathology data.

No.	Gender	Age	Position of glioma	Type	WHO
1	F	22	right temporal lobe	astrocytoma	II
2	M	46	left temporal lobe	astrocytoma	II
3	F	31	right frontal lobe	astrocytoma	II
4	M	23	left thalamus	astrocytoma	II
5	M	55	left temporal lobe	astrocytoma	II
6	F	22	left parietal lobe	astrocytoma	II
7	M	35	left frontal lobe	astrocytoma	II
8	F	36	left temporal lobe	astrocytoma	II
9	M	55	right frontal lobe	astrocytoma	II
10	M	33	right frontal lobe	astrocytoma	II
11	M	59	left insular lobe	astrocytoma	II
12	M	10	right frontal lobe	astrocytoma	II
13	M	56	right frontal-temporal lobe	astrocytoma	II
14	M	61	right frontal-temporal lobe	astrocytoma	II
15	F	40	bilateral frontal-parietal-occipital lobe	astrocytoma	II
16	F	36	left frontal-temporal lobe	astrocytoma	II
17	M	13	right basal ganglia	astrocytoma	II
18	M	19	left frontal lobe/left basal ganglia and septum pellucidum	astrocytoma	II
19	M	14	right frontal lobe	mixed glioma/neuronal tumor	II
20	M	50	left frontal lobe	oligodendro-astrocytoma	II
21	M	32	left frontal lobe	oligodendro-astrocytoma	II
22	M	42	right frontal lobe	oligodendro-astrocytoma	II
23	F	38	right frontal lobe	oligodendro-astrocytoma	II
24	M	35	right frontal lobe	oligodendro-astrocytoma	II
25	M	38	left parietal-occipital lobe	oligodendro-astrocytoma	II
26	M	27	left temporal lobe	oligodendro-astrocytoma	II
27	F	46	right frontal-temporal lobe	oligodendro-astrocytoma	II
28	M	41	left temporal-parietal lobe	oligodendro-astrocytoma	II
29	M	65	left temporal lobe	oligodendro-astrocytoma	II
30	M	34	left temporal lobe	oligodendro-astrocytoma	II
31	M	41	bilateral frontal-parietal lobe	oligodendro-astrocytoma	II
32	F	48	left parietal lobe	oligodendroglioma	II
33	M	44	right frontal lobe	oligodendroglioma	II
34	M	43	left frontal lobe	oligodendroglioma	II
35	M	40	right frontal-parietal lobe	oligodendroglioma	II
36	M	56	right frontal lobe	oligodendroglioma	II
37	F	45	right frontal-insular lobe	anaplastic oligodendro-astrocytoma	III
38	F	58	left frontal-insular lobe	anaplastic oligodendro-astrocytoma	III
39	F	24	right parietal lobe/corpus callosum	anaplastic oligodendro-astrocytoma	III
40	M	40	right frontal-insular lobe	anaplastic oligodendro-astrocytoma	III
41	F	29	right frontal lobe	anaplastic oligodendro-astrocytoma	III
42	M	58	left insular lobe	anaplastic oligodendro-astrocytoma	III
43	F	23	corpus callosum and bilateral frontal-parietal lobe	anaplastic oligodendro-astrocytoma	III
44	F	55	right thalamus	anaplastic oligodendro-astrocytoma	III
45	F	40	left frontal lobe	anaplastic oligodendro-astrocytoma	III
46	M	63	bilateral frontal lobe and corpus callosum	anaplastic oligodendro-astrocytoma	III
47	M	54	left temporal lobe	astrocytoma	III
48	F	53	right frontal lobe and bilateral temporal lobe	astrocytoma	III
49	M	44	right parietal lobe	astrocytoma	III
50	F	42	right temporal lobe	astrocytoma	III
51	F	54	left temporal lobe	astrocytoma	III
52	M	33	right temporal lobe	astrocytoma	III
53	F	52	left thalamus	astrocytoma	III
54	M	34	the third ventricle	glioblastoma	III
55	F	68	right frontal-insular lobe	glioblastoma	IV
56	M	63	left frontal lobe	glioblastoma	IV
57	M	64	left parietal-occipital lobe	glioblastoma	IV
58	F	44	right parietal-occipital lobe	glioblastoma	IV
59	M	40	left temporal lobe	glioblastoma	IV
60	M	48	left frontal-temporal-insular lobe	glioblastoma	IV
61	F	48	right parietal lobe	glioblastoma	IV
62	F	57	left temporal lobe	glioblastoma	IV
63	M	73	left frontal lobe	glioblastoma	IV
64	F	62	left parietal lobe	glioblastoma	IV
65	M	53	left frontal lobe	glioblastoma	IV
66	M	64	right temporal-parietal lobe	glioblastoma	IV
67	M	64	left parietal-occipital lobe	glioblastoma	IV
68	M	49	left parietal lobe/corpus callosum	glioblastoma	IV
69	M	52	bilateral frontal lobe and the knee of corpus callosum	glioblastoma	IV
70	F	45	right frontal lobe	glioblastoma	IV
71	M	60	right parietal-occipital lobe	glioblastoma	IV
72	F	57	left temporal-parietal lobe	glioblastoma	IV
73	M	25	left temporal lobe and left basal ganglia	glioblastoma	IV
74	M	27	left temporal-occipital lobe	glioblastoma	IV
75	F	40	right occipital lobe	glioblastoma	IV
76	F	64	left parietal lobe	glioblastoma	IV
77	M	71	right temporal lobe	glioblastoma	IV
78	F	22	right frontal lobe	gliomatosis cerebri	IV

The scanning parameters of the Siemens 3.0T MR system (Siemens, Germany) were set as follows: gradient field = 40 mT/m, switching rate = 200 T/ms.

T1-weighted: TR = 2 000 ms, TE = 9.8 ms, thickness = 5 mm, space = 6 mm, number of slices = 24, field of view (FOV) = 220×220 mm, each slice has 256×256 pixels.

T2-weighted: TR = 4 500 ms, TE = 84 ms, thickness = 5mm, space = 6 mm, number of slices = 24, FOV = 220×220 mm, each slice has 384×384 pixels.

FLAIR: TR = 7 000ms, TI = 200 ms, TE = 97 ms, thickness = 5 mm, space = 6 mm, number of slices = 24.

MRS: using head coil, 3D multi-voxel chemical shift imaging MRS method, point resolved selective spectroscopy (PRESS) technique, as well as chemical shift selecting saturation (CHESS). TR = 1 700 ms, TE = 30 ms, flip angle = 90 degrees, thickness = 20 mm, volume of interest (VOI) = 80 mm x 80 mm x 20 mm, and one MRS voxel should be 10 mm x 10 mm x 20 mm.

### Procedure of the AQoCE approach

#### A) Pre-processing

The original MRS data consists of two parts: one is header information, which consists of patient information and attributes of spectroscopy such as size of the CSI matrix, vector of position, and length of raw signals. The other is raw signal data in time domain. In this step, we first denoise the raw signal by apodization, then obtain the spectrum of the denoised signal by Fast Fourier Transformation (FFT).

#### B) Convex-envelope based baseline fitting

In the spectrum, the signal intensity of water is often several orders of magnitude larger than the signal intensities of other metabolite components. The residual water peak tails are often considered as an additional baseline with fluctuation. It is usually assumed that the baseline is a very smooth curve and each metabolite component peak is in a narrow range, so the baseline can be approximated by a piecewise linear curve. In this study we chose the convex envelope for fitting.

Let *f*{*n*} denote a certain range of the whole spectrum, *h*{*n*} is the convex envelope of *f*{*n*}, *k* is the length of *f*{*n*}, and *h*{*n*}. *h*{*n*} should be the maximum of a sequence set which has the property *g*{*n*} as described in Eq (1).

$$D=\left\{f(n)\geq g(n)\;\forall n=1,\ldots ,k\;|\;g(\frac{n_{1}+n_{2}}{2})\leq \frac{g(n_{1})+g(n_{2})}{2}\;\forall n_{1},n_{2}=1,\ldots ,k\right\}$$ (1)

The convex envelope sequence *h*{*n*} is generated as follows:

Let *h*_*i*_{*n*} denote the convex envelope of the first *i+2* points of *f*{*n*}, *I*_*i*_{*n*} is the index set of all points in *h*_*i*_{*n*}. Initialize *i* to be 1, then we have

$$h_{1}\{n\}=\left\{f\left(1\right),f\left(2\right),f\left(3\right)\right\},\;I_{1}\{n\}=\left\{1,2,3\right\}$$ (2)

Check if the following inequality is held or not:

$$h_{i}(k-2)\left(I_{i}(k)-I_{i}(k-1)\right)+h_{i}(k)\left(I_{i}(k-1)-I_{i}(k-2)\right)>h_{i}(k-1)\left(I_{i}(k)-I_{i}(k-2)\right)$$ (3)

Where *k* is the length of *h*_*i*_{*n*}. If the inequality is not held, remove point *h*_*i*_(*k−*1) and its index *I*_*i*_(*k−*1) from *h*_*i*_{*n*} and *I*_*i*_{*n*}, respectively. As a result, the length of *h*_*i*_{*n*} and *I*_*i*_{*n*} is reduced by 1. Then check the inequality again until it is held.

If *i* +2 = *k*, *h*_*i*_{*n*} is already the convex envelope of *f*{*n*}. Go to step *d*. If *i* +2 < *k*, then initialize *h*_*i+1*_{*n*} and *I*_*i+1*_{*n*} as Eq (4) and increase *i* by 1. Go back to step *b* to make sure of the convex property.

$$h_{i+1}\{n\}=\left\{h_{i}\{n\},f\left(i+3\right)\right\},\;I_{i+1}\{n\}=\left\{I_{i}\{n\},\;i+3\right\}$$ (4)

Restore removed points by linear interpolation, and the generation of the convex envelope sequence is complete.

#### C) Bias correction

Use the method described in B) to generate the convex envelope for NAA (2.0ppm ± 0.5ppm), which is one of the most significant metabolite component, that we model on only one peak for simplicity and robustness, then subtract the convex envelope from the spectrum, and the maximum point in this range can be regarded as the actual position of the NAA peak. Shift the spectrum so that the actual position of the NAA peak is located at 2.02ppm. This is the aligned spectrum.

#### D) Sectional baseline removal

Because ranges of some metabolite components were overlapping, we generated the convex envelopes for three sections. These sections are 4.3 ppm to 3.2 ppm (Cho), 3.9 ppm (Glx) to 2.0 ppm (NAA), 3.0 ppm (Cr) to 1.5 ppm, and 1.7 ppm to 1.0 ppm. Let *h*_*1*_(*x*), *h*_*2*_(*x*), *h*_*3*_(*x*) and *h*_*4*_(*x*) be their convex envelopes. Then we define the whole baseline approximation *h*(*x*) as:

$$h(x)=\{\begin{array}{c} h_{1}(x)\;4.3\geq x>3.9\\ \text{min}(h_{1}(x),h_{2}(x))\;3.9\geq x>3.2\\ h_{2}(x)\;3.2\geq x>3.0\\ \text{min}(h_{2}(x),h_{3}(x))\;3.0\geq x>2.0\\ \begin{array}{l} \;h_{3}(x)\;2.0\geq x>1.7\\ \text{min}(h_{3}(x),h_{4}(x))\;1.7\geq x>1.5\\ \;h_{4}(x)\;1.5\geq x>1.0 \end{array} \end{array}$$ (5)

Subtracting *h*(*x*) from the aligned spectrum, the residual spectrum becomes meaningful and clear.

#### E) Metabolite peak detection

In the range of theoretical position ±0.1ppm for every metabolite component of interest, take the maximum point as the peak value.

As this approach only requires a linear scan of the spectrum which makes it run in linear time, we can efficiently analyze a multi-voxel spectrum and obtain a metabolite component peak value.

### Method of analysis of metabolite components

#### A) ROI selection

For each case, two neuroimaging experts with years of experience made the following selections:

Determine the location of the CSI slice. Normally, it is the one slice of an axial image which has a maximum volume of tumor.

Put three regions of interest (ROI) in the enhanced tumoral area, peritumoral area which is about 1 cm from tumor margins, and contralateral areas distant to the tumor.

In each ROI, choose 1~3 MRS voxels to measure according to the tumor size.

Use the average to reduce the error. Finally, normalize the results of the two experts and also obtain the average.

#### B) Relative quantitative calculation of metabolites

**Fig 1:**
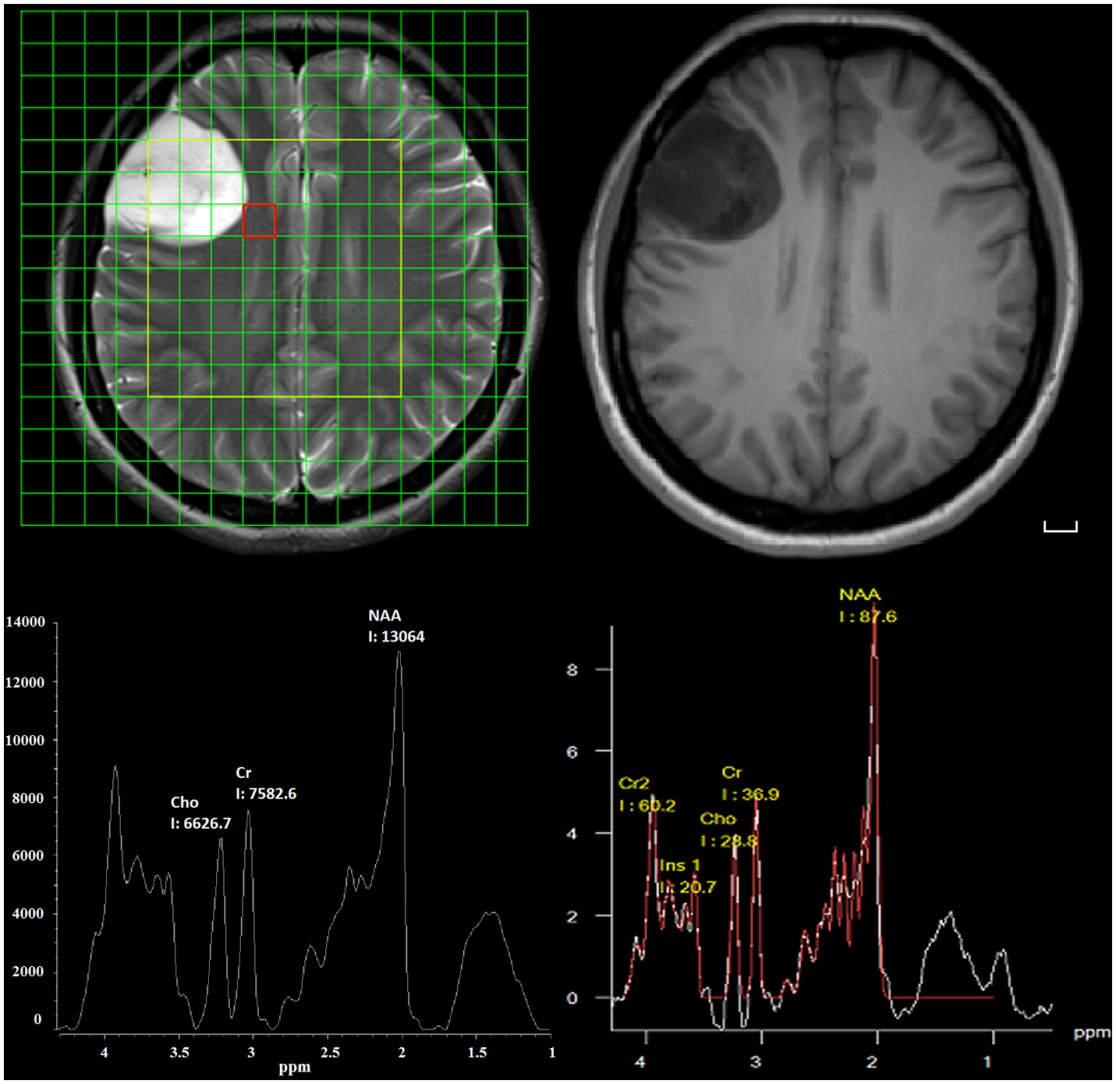
Images of a 31-year-old female with a grade II astrocytoma. MRS grid map overlaid on a T2-weighted image (top left). T1-weighted image (top right). Spectroscopy processed by the AQoCE approach (bottom left). Spectroscopy processed by Siemens processing software (bottom right). The chosen spectroscopy volume of interest (VOI) is located in the peritumoral area. Scale bar: 1 cm.

**Fig 2:**
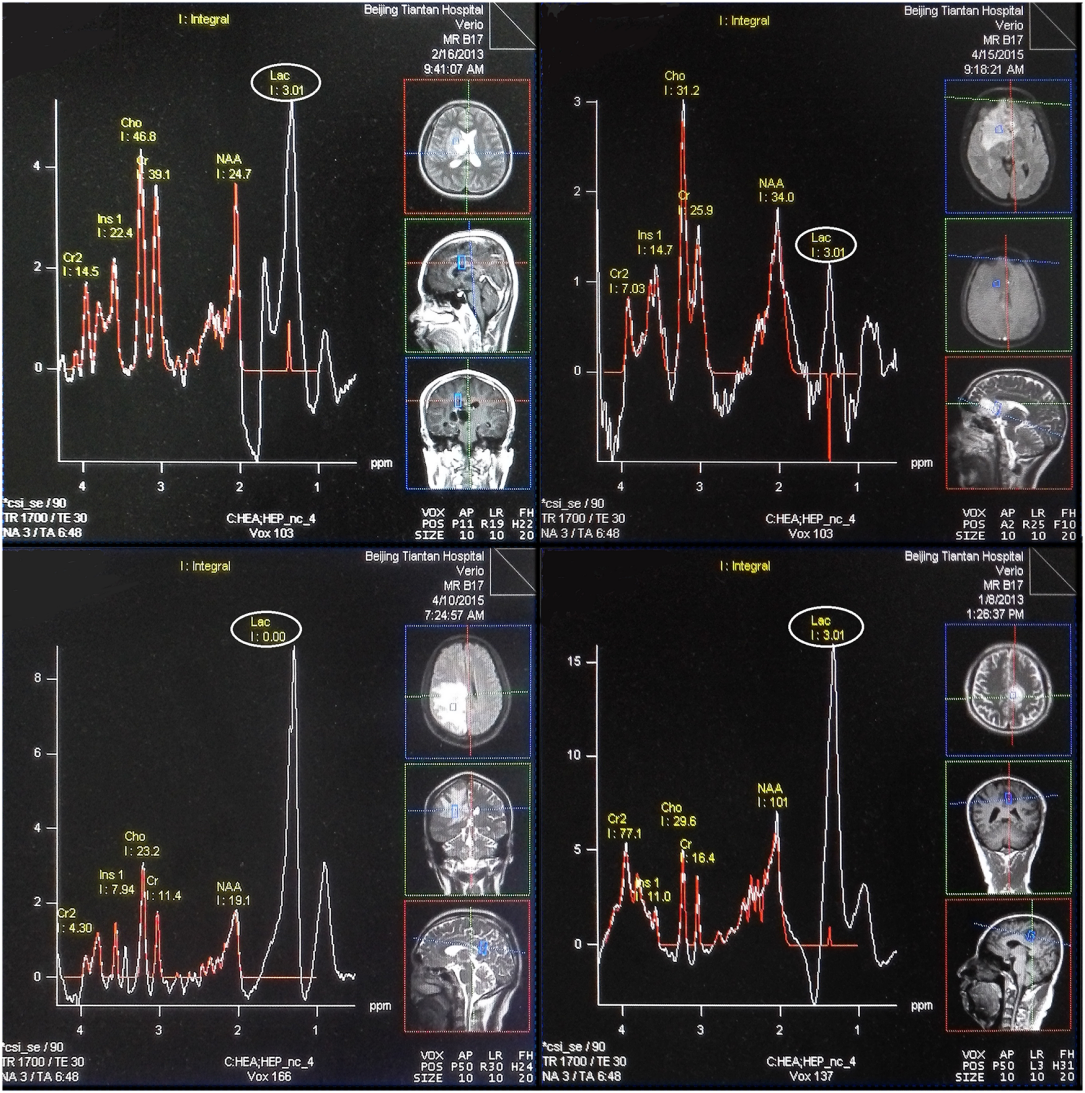
Samples of spectroscopy processed by Siemens processing software with LL quantification error. All the chosen spectroscopies belong to tumoral area. The quantification results are presented in red line, and these errors should be found at the location of Lac point by comparing with original spectrum (white line).

The metabolites Cho, NAA, Cr, and sum of Lipid and Lac (Lip-Lac, LL) were acquired in short TE spectrum. The raw data were processed using two methods: the convex-envelope based automated quantitative approach (AQoCE) and Siemens MRS data processing software (Fig 1). Metabolic ratios (Cho/NAA, Cho/Cr and LL/Cr) were used in the statistical analysis. For reasons of quantification errors in 29/78 of Siemens LL results by modeling (Fig 2), and no quantification error in AQoCE LL results by taking the maximum, only the ratio of LL/Cr calculated by AQoCE is used.

#### C) Statistical analysis

Our design for statistical tests is presented here. All statistical tests are two-sided, performed with SPSS19.0 (SPSS Inc., Chicago, IL). *p* values less than 0.05 were considered statistically significant.

First, an independent sample *t*-test was used for evaluating significant difference between the metabolic ratios in high- and low-grade gliomas, including Cho/NAA, Cho/Cr and LL/Cr.

Second, in order to determine the sensitivity and specificity of AQoCE, post-processing methods were used for identifying glioma grade and the receiver operating characteristic curve (ROC). To determine the metabolites which could be significant as diagnostic indicators, Cho/Cr, Cho/NAA and LL/Cr were chosen. Furthermore, logistic regression which combines these ratios, provided better predictive value.

Third, as the data distribution was skewed, the Kruskal-Wallis test followed by all pairwise multiple comparisons was used for evaluating the significance between the affected (tumoral, peritumoral area) and contralateral area.

## Results

In this study, the default MRS data processing software, run on a Siemens Trio Tim 3.0T MRI’s workstation, was selected as a reference approach and was called “Siemens” in the present study. “Siemens” was used to evaluate the performance of the proposed AQoCE method. Both AQoCE and “Siemens” were used to analyze all 78 cases, including 36 cases of LGG and 42 cases of HGG: 18 astrocytoma (WHO II), 5 oligdendroglioma (WHO II), 12 oligdendro-astrocytoma (WHO II), 1 mixed neuro-glioma (WHO II), 10 anaplastic oligdendro-astrocytoma (WHO III), 7 astrocytoma (WHO III), 24 glioblastoma (WHO IV), 1 gliomatosis cerebri (WHO IV). The results are as follows.

First, ^1^H-MRS is valuable in the clinical diagnosis and prognosis (by identifying the glioma grade). Gliomas consist of abnormal glial cells proliferation. During growth, tumor cells infiltrate and push normal neurons, resulting in a slight decrease in Cr and NAA, a significant increase in Cho, and an appearance of Lac and Lipid [23], as both approaches demonstrate. In our study, the AQoCE approach demonstrates that the ratios of Cho/NAA, Cho/Cr and LL/Cr are significantly different between LGG and HGG (*p* = 0.005, *p* = 0.002 and *p* = 0.001, respectively). The “Siemens” approach demonstrates only that the Cho/Cr ratio shows a significant difference (*p* = 0.007). Brain tumor ratios of Cho/NAA, Cho/Cr and LL/Cr are positively correlated with brain glioma grading. They can help to differentiate high- from low-grade gliomas, which is in agreement with other reports [24]. Details are shown in Tables 2 and 3.

**Table 2:** Statistics of intratumoral metabolic ratios by AQoCE and Siemens approaches.

approach	low-grade gliomas (LGG)			high-grade gliomas (HGG)		
	Cho/NAA	Cho/Cr	LL/Cr	Cho/NAA	Cho/Cr	LL/Cr
**Siemens**	0.95±0.58	1.31±0.49	-	1.12±0.56	2.59±2.87	-
**AQoCE**	1.04±0.43	1.27±0.31	1.07±1.32	1.35±0.50	1.70±0.76	2.65±2.51

Note: Data are expressed as mean±standard deviation.

**Table 3:** *t*-test of intratumoral metabolic ratios in AQoCE and Siemens approaches.

approach	Cho/NAA	Cho/Cr	LL/Cr
**Siemens**	1.314/0.193	2.841/0.007	-
**AQoCE**	2.870/0.005	3.251/0.002	3.552/0.001

Note: Data are *t*/*p* values of independent sample *t*-test.

Second, the ROC curve demonstrates that the AQoCE approach predicts the grade of gliomas by combining Cho/NAA, Cho/Cr and LL/Cr in the tumoral area with good performance. The logistic regression formula used for combination of metabolic ratios is:

$$probabilities\;=1/[1+e^{-\left(-a\;+\;b\;\times \text{ Cho}/\text{Cr }+\;c\;\times \text{ Cho}/\text{NAA + }d\;\times \text{ LL}/\text{Cr}\right)}.$$ (6)

*a* = 4.733, *b* = 1.580, *c* = 1.290, *d* = 0.698 are obtained by fitting to the results of AQoCE approach. The best diagnostic cut-off point for the probabilities of logistic regression is 0.376. In this setting, the AQoCE approach achieves a sensitivity of 92.9%, a specificity of 72.2%, and an area under the ROC curve (AUC) of 0.860. While fitting to the results of “Siemens” approach, *a* = 3.168, *b* = 1.902, *c* = 0.252 are obtained. The best cut-off point is 0.446, with a sensitivity of 78.6%, a specificity of 75.0%, and an AUC of 0.806. The ratio of Cho/NAA achieves little for the prediction because it shows no significant difference in “Siemens” approach. See Table 4 for details.

**Table 4:** Results of intratumoral ROC using the AQoCE and Siemens approaches.

**AQoCE**	**cut-off point**	**Sensitivity (%)**	**Specificity (%)**	**AUC**
**Cho/NAA**	1.118	69.0	66.7	0.687
**Cho/Cr**	1.339	81.0	63.9	0.774
**LL/Cr**	1.728	61.9	91.7	0.802
**combination**	0.376	92.9	72.2	0.860
**Siemens**	**cut-off point**	**Sensitivity (%)**	**Specificity (%)**	**AUC**
**Cho/NAA**	0.902	66.7	61.1	0.616
**Cho/Cr**	1.422	78.6	75.0	0.803
**combination**	0.446	78.6	75.0	0.806

Note: The combination of metabolic ratios is established by the logistic regression formula. AUC: Area Under Curve.

Third, in theory, along with a sort order from tumoral area to peritumoral area and contralateral area, both Cho/NAA and Cho/Cr ratios show a regular variation from high to low. In this study, we analyzed the Cho/NAA and Cho/Cr in the tumoral area, peritumoral area and contralateral area. The results show that both Cho/NAA and Cho/Cr in the AQoCE approach demonstrates a significant difference, with *p*≤0.019 between all three areas. For the “Siemens” approach, the ratios Cho/NAA and Cho/Cr demonstrate a significant difference only between the tumor and other areas. In this respect, the AQoCE approach is more sensitive with the tumor margin region. Results are shown in Tables 5 and 6.

**Table 5:** Statistics of metabolic ratios in different areas using AQoCE and Siemens approaches.

	intratumor		peritumor		contra lateral areas	
approach	Cho/NAA	Cho/Cr	Cho/NAA	Cho/Cr	Cho/NAA	Cho/Cr
**Siemens**	1.04±0.57	2.00±2.21	0.54±0.24	0.97±0.23	0.45±0.18	0.87±0.21
**AQoCE**	1.21±0.49	1.50±0.63	0.59±0.16	0.95±0.16	0.47±0.11	0.84±0.16

Note: Data are expressed as mean±standard deviation.

**Table 6:** Kruskal-Wallis test of metabolic ratios in different areas using AQoCE and Siemens approaches.

	intratumor / peritumor		intratumor / contra lateral areas		peritumor / contra lateral areas	
approach	Cho/NAA	Cho/Cr	Cho/NAA	Cho/Cr	Cho/NAA	Cho/Cr
**Siemens**	6.946/0.000	7.009/0.000	9.107/0.000	9.313/0.000	2.161/0.092	2.304/0.064
**AQoCE**	7.824/0.000	7.683/0.000	11.526/0.000	10.405/0.000	3.702/0.001	2.722/0.019

Note: Data are *h*/*p* values of the Kruskal-Wallis test.

## Discussion

An overview of the recent literatures on brain tumor diagnosis using an *in vivo* ^1^H-MRS quantitative method shows that most authors presented the statistical analysis results for tumor type-specific or for tumor grading based on ^1^H-MRS quantitative results using MRI application software provided by GE, Siemens and Philips [25–32]. Their various results imply that although ^1^H-MRS can be used as an auxiliary tool for brain tumor diagnosis, the MRS data processing methods are not robust enough for *in vivo* complex tissues. For example, model-based methods such as HLSVD are perfect for phantom data, but not sufficient for *in vivo* glioma data [33]. This means that the MRS quantitative approach is crucial for obtaining the content of metabolites, which can directly impact the accuracy of diagnosis. This is particularly true for the method of residual water removal, baseline estimation and metabolite quantification.

The process of the “Siemens” reference approach includes water suppression, filtering, filling zero, Fourier transformation, frequency correction, phase correction, baseline correction and curve fitting, with a total of 8 steps. In contrast, the process of the AQoCE approach includes apodization and Fourier transformation, convex-envelope based baseline fitting, bias correction, sectional baseline removal, peak detection and modelization, 5 steps in all. The main innovation is the convex-envelope based baseline fitting method, taking the lower enveloping curve as baseline which is achieved by feature-point extraction and linear interpolation.

This study highlights some advantages of AQoCE:

1. Versatile: No specific parameter is needed in advance and the metabolic components of *in vivo* and complex tissues can be better represented, due to the assumption that the tails of residual water fit the convex envelope curve.
2. Efficient: Much faster runtime than many existing methods. It takes only a few seconds to process one patient’s data by AQoCE, while an example of HLSVD exam takes minutes.
3. Significant: The quantitative results are statistically significant for separating three types of voxels (tumoral, peritumoral area and contralateral area) and in distinguishing between LGG and HGG.

Moreover, the proposed analysis method of metabolic components, including ROI selection, relative quantitative calculation of metabolites, and statistical methods of analysis is reasonable and valid.

As current research reports on glioma grading show, MRS quantitative analysis may be the simplest, safest, and most effective method. However, the accuracy is not as high as the combination analysis of MRS and PWI [32, 34–36].

There are still some limitations to this study. First, the sample size is not large enough to cover all types and grades of tumors and samples are heterogeneous in terms of WHO grade, which lessens statistical power. We cannot completely rule out the possibility of changes in results when the sample size increases. In the future, we will increase sample size and complete clinical data. Second, choosing ROI accurately and reasonably can greatly impact the metabolites. This process should be more formally established to avoid errors. Third, some important metabolites have not been taken into account, such as myo-Inositol (mI) [37]. It would be of value to analyze their characteristics and evaluate their diagnostic value based on obtained metabolic data in order to improve the diagnostic and prognosis accuracy for gliomas. Finally, apply the new approach on combinations with other new magnetic resonance imaging technologies such as PWI, for higher specificity and sensitivity in glioma screening [38–39].

An automated CSI data processing software that implements AQoCE is shown in S1–S3 Figs. The user only needs to load CSI raw data acquired by the Siemens MR system (with the extension of.rda) and click on any voxel of interest or select any number of voxel of interest, then all of the selected multi-voxel MRS results, both wave form and peak value will be immediately displayed in a new window.

## Conclusions

In this work, we propose a new automated quantitative approach based on a convex envelope (AQoCE) and apply it to multi-voxel ^1^H-MRS analysis. Under the limited clinical data used, this approach can efficiently evaluate brain glioma grading and demonstrate characteristics of brain glioma metabolism. It can also suggest the presence of peritumoral area infiltration. AQoCE has been integrated into our MRS data processing software and will be available as a free download on the following website: http://mdpslab.voog.com/softwares.

## Supporting Information

S1 Fig Plot view mode of the MRS processing software. One or multiple spectroscopies are shown in right window.
(TIF)

S2 Fig Grid view mode of the MRS processing software. Multiple grids of spectroscopy can be chosen by mouse drag. Thumbnail of spectroscopy is shown in right window.
(TIF)

S3 Fig Spectrogram view mode of the MRS processing software. Multiple grids of metabolic ratios can be chosen by mouse drag and drop. Color bar is used as the indication of the ratio intensity.
(TIF)

S1 Table Pathology information and data acquired by AQoCE & Siemens approach. All the data used in the statistical analysis is included in the file.
(XLSX)
