# Supplementary figures and images for: Convex-Envelope Based Automated Quantitative Approach to Multi-Voxel 1H-MRS Applied to Brain Tumor Analysis
Source: PLoS One. 2015 Sep 14;10(9):e0137850. doi: 10.1371/journal.pone.0137850 (PMC4569259; doi:10.1371/journal.pone.0137850)

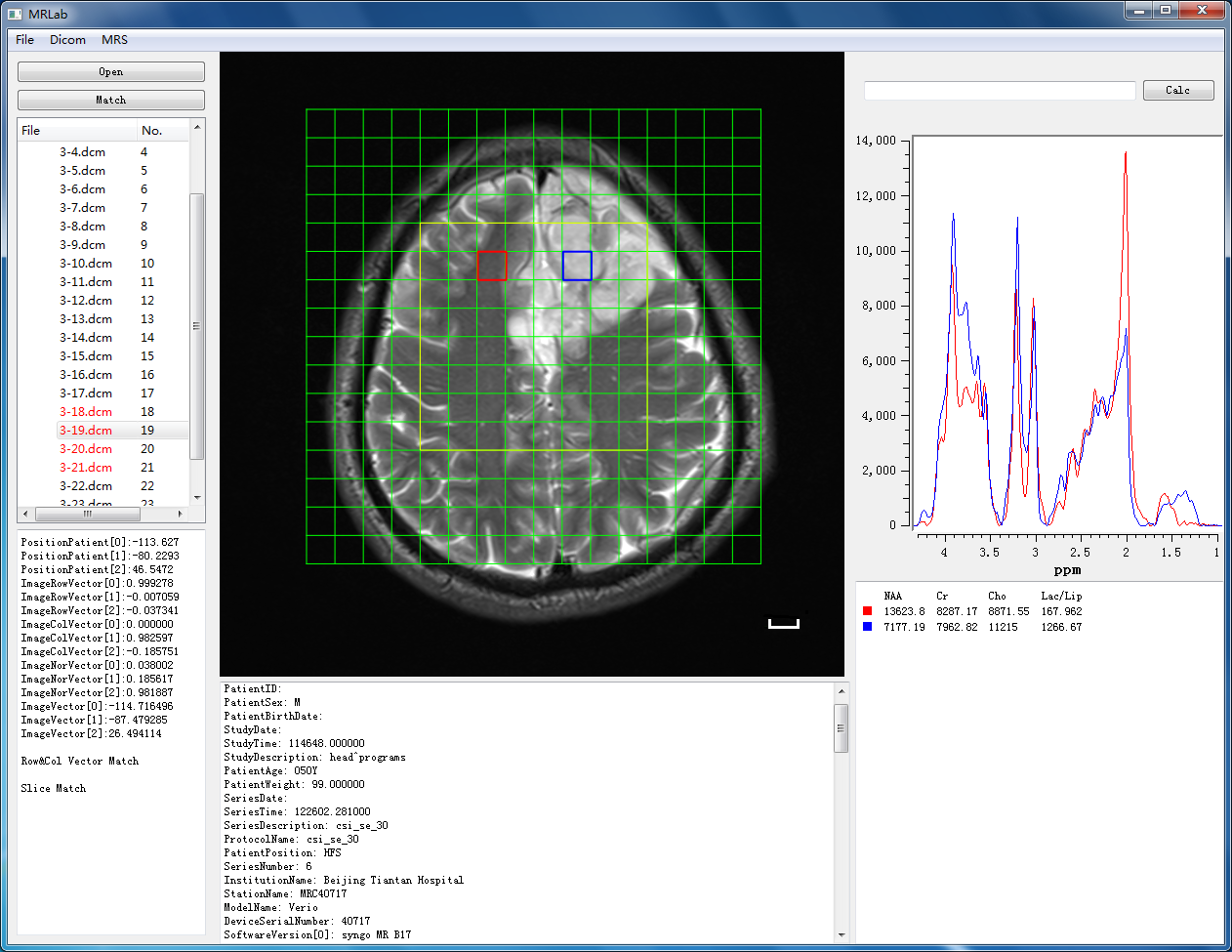

Supplement: S1 Fig — One or multiple spectroscopies are shown in right window. (TIF) [file pone.0137850.s001.tif]

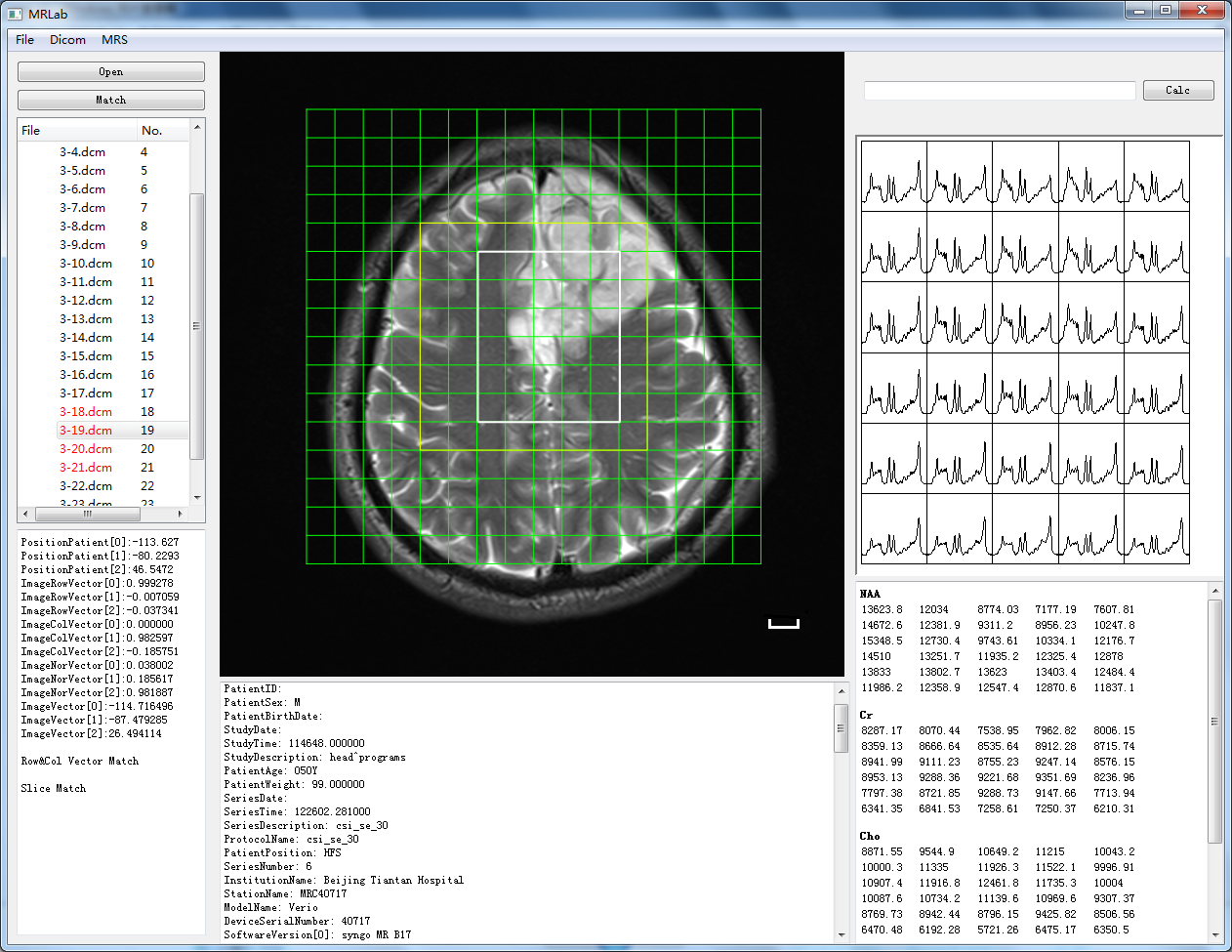

Supplement: S2 Fig — Multiple grids of spectroscopy can be chosen by mouse drag. Thumbnail of spectroscopy is shown in right window. (TIF) [file pone.0137850.s002.tif]

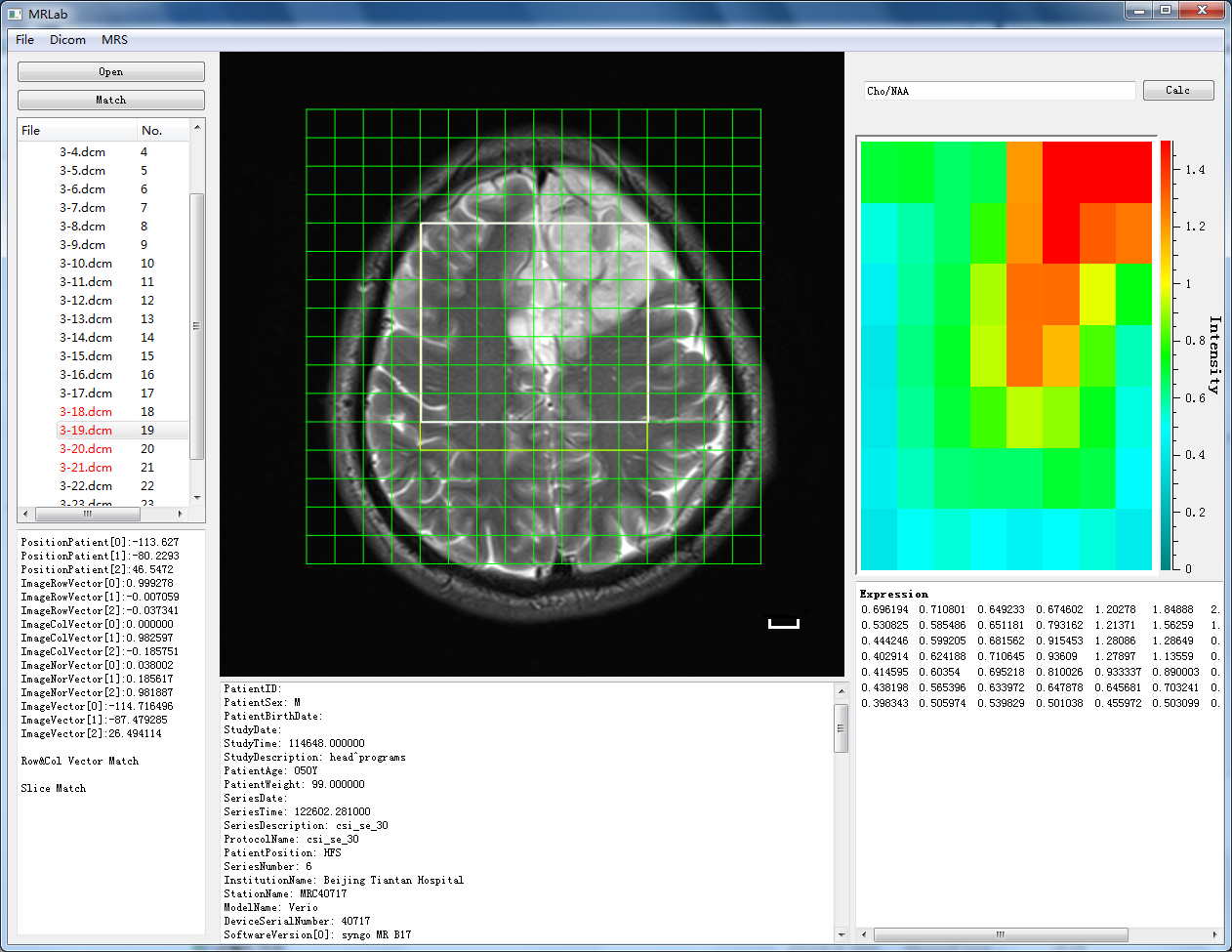

Supplement: S3 Fig — Multiple grids of metabolic ratios can be chosen by mouse drag and drop. Color bar is used as the indication of the ratio intensity. (TIF) [file pone.0137850.s003.tif]
